# Phenotypic characteristics of human type II alveolar epithelial cells suitable for antigen presentation to T lymphocytes

**DOI:** 10.1186/1465-9921-12-15

**Published:** 2011-01-24

**Authors:** Véronique Corbière, Violette Dirix, Sarah Norrenberg, Mattéo Cappello, Myriam Remmelink, Françoise Mascart

**Affiliations:** 1 Laboratory of Vaccinology and Mucosal Immunity, Université Libre de Bruxelles (U.L.B.), Brussels, Belgium; 2 Laboratory of Pathology, Hôpital Erasme, Université Libre de Bruxelles, Brussels, Belgium; 3 Department of Thoracic Surgery, Hôpital Erasme, Université Libre de Bruxelles, Brussels, Belgium; 4 Immunobiology Clinic, Hôpital Erasme, Université Libre de Bruxelles, Brussels, Belgium

## Abstract

**Background:**

Type II alveolar epithelial cells (AECII) are well known for their role in the innate immune system. More recently, it was proposed that they could play a role in the antigen presentation to T lymphocytes but contradictory results have been published both concerning their surface expressed molecules and the T lymphocyte responses in mixed lymphocyte cultures. The use of either AECII cell line or fresh cells could explain the observed discrepancies. Thus, this study aimed at defining the most relevant model of accessory antigen presenting cells by carefully comparing the two models for their expression of surface molecules necessary for efficient antigen presentation.

**Methods:**

We have compared by flow cytometry the surface expression of the major markers involved in the immunological synapse on the A549 cell line, the most popular model of type II alveolar epithelial cells, and freshly isolated cells. HLA-DR, CD80, CD86, ICOS-L, CD54, CD58 surface expression were studied in resting conditions as well as after IFN-γ/TNF-α treatment, two inflammatory cytokines, known to modulate some of these markers.

**Results:**

The major difference found between the two cells types was the very low surface expression of HLA-DR on the A549 cell line compared to its constitutive expression on freshly isolated AECII. The surface expression of co-stimulatory molecules from the B7 family was very low for the CD86 (B7-2) and ICOS-L (B7-H2) and absent for CD80 (B7-1) on both freshly isolated cells and A549 cell line. Neither IFN-γ nor TNF-α could increase the expression of these classical co-stimulatory molecules. However CD54 (ICAM-1) and CD58 (LFA-3) adhesion molecules, known to be implicated in B7 independent co-stimulatory signals, were well expressed on the two cell types.

**Conclusions:**

Constitutive expression of MHC class I and II molecules as well as alternative co-stimulatory molecules by freshly isolated AECII render these cells a good model to study antigen presentation.

## Background

Type II alveolar epithelial cells (AECII) are since long recognized as important players of the innate immune system, secreting antimicrobial proteins like surfactant protein A, C and D, but also producing a variety of cytokines and chemokines [1-3]. Due to their location, they are exposed to microbes reaching the alveolus and can be infected by several infectious agents, such as influenza virus, severe acute respiratory syndrome-coronavirus, *Legionella pneumophila, Bacillus anthracis *or *Mycobacterium tuberculosis *which is well known to multiply and to survive within AECII [4-10]. Indeed alveolar epithelial cells are being by far more numerous than the macrophages, the phagocytic cell prototype [11]. However besides the AECII, the alveolar surface is also covered by type I AEC but these cells mostly play a role for gaz exchange [12]. In contrast, cuboidal AECII were suggested to play a possible role of non-professional antigen-presenting cells as they were reported to express both class I and class II major histocompatibility complex molecules (MHC) [13]. Interestingly, AECII are in contact with a huge amount of lymphocytes, the cells involved in the development of specific immune responses. Indeed, the number of lymphocytes in the lung interstitium has been reported to be 10^10^, which is similar to the number of circulating lymphocytes [14].

Many studies turned therefore to a better characterization of the AECII phenotype and more precisely on the detection of surface molecules involved in antigenic presentation. As T-cell receptor engagement and co-stimulatory signals are usually required for the full activation of T cells, different authors have analyzed the expression of co-stimulatory molecules by AECII. However, several contradictory results were published, each paper focusing on a limited amount of phenotypic markers. Major differences between the results might be explained by technical differences between the studies [13,15-19]. In addition, most studies were performed on a human tumor cell line, the A549, defined as a model of human AECII [20], as freshly isolated AECII from human pulmonary pieces are rather difficult to obtain.

The aim of the study was to compare both models and to define the most suitable one to study antigen presentation. In this paper, we report a detailed phenotypic analysis of human AECII comparing the human tumor cell line A549 to freshly isolated human AECII. We have characterized the expression of MHC-class II molecules and the expression of different co-stimulatory molecules known to be involved in the immunological synapse, CD80, CD86, ICOS-L, CD40, CD54, CD58. The expression of these molecules was analyzed first on resting cells and then on cytokine-activated cells. To mimic inflammation, we chose to analyze the effect on the AECII phenotype of two major inflammatory cytokines, IFN-γ and TNF-α, known to modulate some of these surface molecules [17,21-26].

## Methods

### A549 cell line culture

A549, a human alveolar type II epithelial cell line from an adenocarcinoma (LGC Promochem, UK/ATCC^®^; Number: CCL-185™) was maintained in Dulbecco's modified Eagle's medium (DMEM, LONZA, Verviers, Belgium) supplemented with 10% heat-inactivated foetal calf serum (FCS, PAA Laboratories GmbH, Pashing, Austria) at 37°C in a 5% CO2 atmosphere. For the experiments, the cell line was used from the 5^th ^to the 13^th ^passage.

### Human pulmonary type II alveolar epithelial cells

After ethical committee agreement (Comité d'Ethique-Hôpital Erasme, reference number P2007/175), AECII were isolated from macroscopically tumor free regions of lung tissues obtained following lobectomy or pneumectomy for lung cancer. The AECII isolation was adapted from a previously described technique [27]. After differential adherence of contaminating mononuclear cells, non-adherent AECII were plated at 5 × 10^5 ^cells/well in 48 wells flat-bottomed plates precoated with type I collagen (1%) to obtain pure AECII [28]. Cells reached confluence after 24 or 48 hours. The purity averaged 85.78% (81.78% - 90.03%) (median, inter-quartile ranges) for the nine independent AECII isolations as assessed by flow cytometry (see below).

### Flow cytometry

This technique was used to assess the purity of the cell suspensions and to characterize their phenotype after cytokine stimulation. Cells were first incubated for 30 minutes with FCS before staining to avoid non specific fixation of the antibodies.

To assess the purity of cell suspensions, cells were stained with antibodies directed to surface molecules not expressed by AECII: anti-human CD19-FITC (clone 4G7, mouse IgG1 kappa), CD45-PerCp (clone 2D1, mouse IgG1 kappa), CD11b-APC (clone D12, mouse IgG2a kappa), CD11c-APC (clone S-HCL-3, mouse IgG2b kappa) and CD14-APC (clone MphiP9, mouse IgG2b kappa). A goat polyclonal IgG anti-human DC-LAMP-PE (CD208) was used to identify AECII in the cell suspension as DC-LAMP is constitutively expressed by AECII [29,30]. DC-LAMP staining was performed after fixation and permeabilization of the cells (lysing solution and permeabilization solution, BD Biosciences). All reagents were obtained from BD Biosciences (Erembodegem, Belgium), except the antibody to DC-LAMP which was from R&D Systems Europe (Abingdon, UK).

To characterize the phenotype of the A549 cell line and of primary AECII, the cells were stained with anti-human antibodies to HLA-DR-PE (clone L243, mouse IgG2a kappa), CD80-PE (clone L307.4, mouse IgG1 kappa), CD86-PE (clone FUN-1, mouse IgG1 kappa), ICOS-L (clone 2D3/B7-H2, mouse IgG2b kappa), CD40-FITC (clone 5C3, mouse IgG1 kappa), CD54-APC (clone HA58, mouse IgG1 kappa), CD58-FITC (clone 1C3, mouse IgG2a kappa). All the antibodies used were obtained from BD Biosciences.

Flow cytometric analysis was performed using a FACSCanto II (Becton Dickinson) and the FlowJo software (Tree Star, Ashland, OR, USA). Both the percentages of positive cells and the median of fluorescence intensity (MFI) were evaluated for each triplicate. For the MFI analysis, specific fluorescence intensity variations observed after activation were determined by the ratio between the MFIs of stimulated cells (MFIs) and resting cells (MFIr). As an increase in autofluorescence was observed during short-term cultures, the value obtained for non-labelled cells (MFIx 0) was subtracted from the MFI of the labelled cells (MFIx), for both resting and stimulating cells (MFIr and MFIs): (MFIs - MFIs 0)/(MFIr - MFI r 0)

### Cytokine stimulations

Cells were plated in 48 wells flat-bottomed plates (A549: 5 × 10^4 ^cells/well; AECII: 5 × 10^5 ^cells/well), and incubated until confluence (A549: 22 hours; AECII: 24/48 hours). In parallel to resting conditions, the cells were *in vitro *stimulated with human recombinant IFN-γ (100 ng/ml), human recombinant TNF-α (50 ng/ml) or a combination of the two cytokines (IFN-γ 50 ng/ml and TNF-α 25 ng/ml) (R&D Systems Europe) during 24 hours before analyzing their phenotype by flow cytometry. Seven and four independent experiments were performed in triplicate for A549 cell line and AECII respectively.

### Statistics

Results are presented as mean values obtained from triplicate and medians were used to compare groups. The non-parametric Mann-Whitney test was applied to compare phenotypic markers expression of A549 versus AECII in resting conditions. To compare the percentages of positive cells after cytokine stimulations versus resting conditions, the non-parametric Kruskal-Wallis test combined with the Dunn's multiple comparison test was used. The non-parametric Kruskal-Wallis test associated with the Dunn's multiple comparison test was used to compare MFI after stimulating conditions versus resting condition. A value of *P <*0.05 was considered to be significant. All results were obtained with the GraphPad Prism version 4.00 for Windows (GraphPad Software, San Diego, CA, USA, http://www.graphpad.com).

## Results

### Phenotypic characterization of freshly isolated type II AEC compared to the A549 cell line

**Figure 1:**
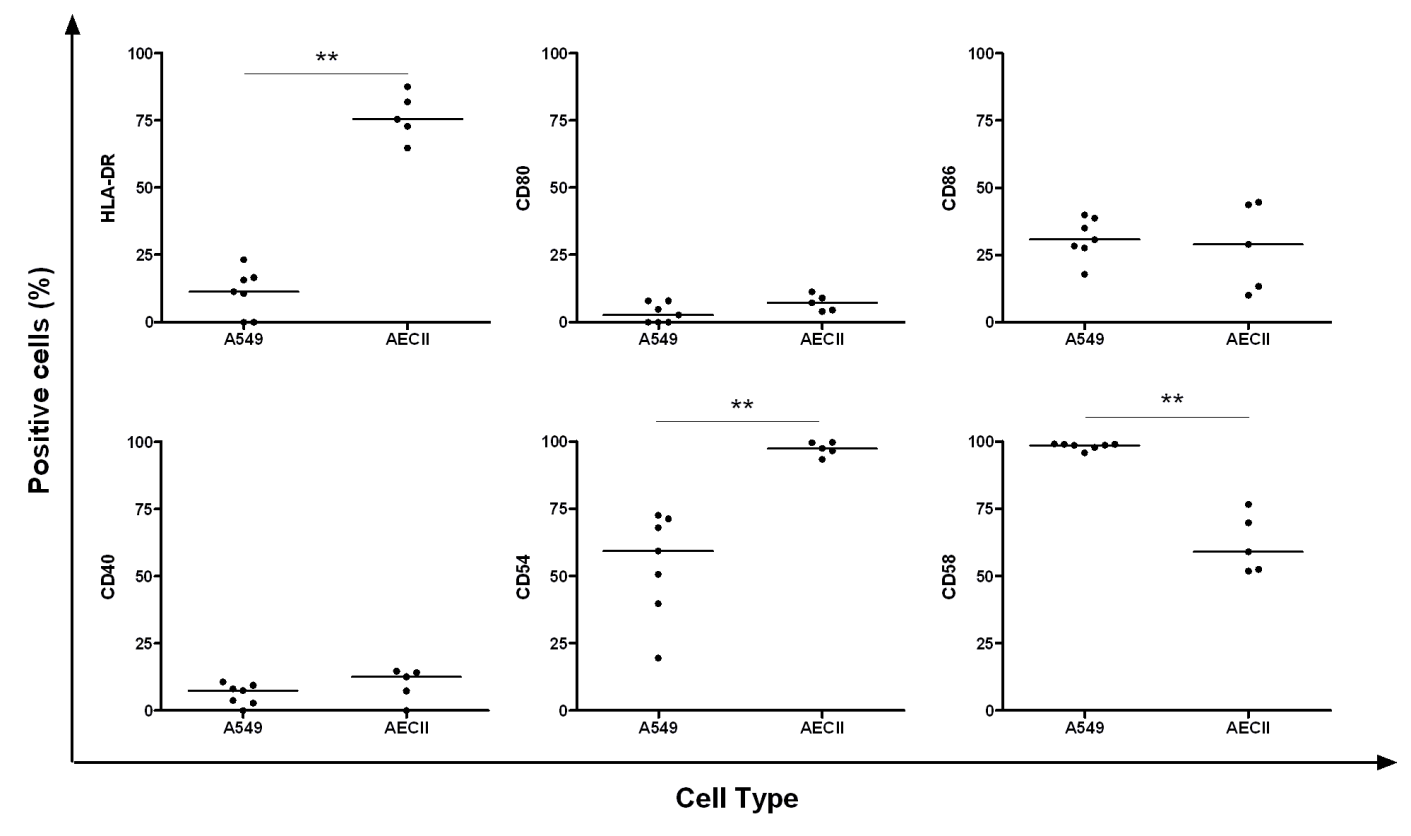
**Phenotypic characterization by flow cytometry of A549 cells compared to freshly-isolated AECII in resting conditions**. A549 cell line and fresh isolated AECII were cultured until confluence and stained with the indicated surface antibodies for phenotypic analysis by flow cytometry. Each experimental condition (seven for A549 cell line and five for AECII) was performed in triplicate, the mean value being represented by a dot. Medians are represented as horizontal bars. A value of *P <*0.05 was considered to be significant. ** *P *< 0.01.

Type II AEC isolated from human lung cultured until confluence were stained with phenotypic markers and compared to the A549 cell line, often used as a model of human AECII. The cells were stained with anti-human antibodies to HLA-DR, as it is the most strongly expressed class II locus. Whereas a high proportion of freshly isolated AECII expressed at their surface HLA-DR molecules (median 75.44%, ranges: 68.73% - 84.68%), only a minority of A549 cells did express this marker (11.40%, 0.03% - 16.60%), resulting in significant differences in the expression of MHC class II molecules between the two types of cell suspensions (*P *< 0.01) (Figure 1).

Analysis of the expression of the co-stimulatory molecules from the B7 family indicated that few cells of both suspensions express these markers. The study of the fresh AECII showed almost no expression of CD80 (7.22%, 4.26% - 10.14%), a low expression of CD86 (28.93%, 11.74% - 44.13%) and of ICOS-L (14.82%, 8.32% - 16.76%). Similar results were obtained for the cell line A549 (Figure 1 and data not shown for ICOS-L). The CD40 molecule was expressed only on a very low proportion of epithelial cells, respectively on 12.54% of the AECII (3.66% - 14.41%) and on 7.37% of the A549 cells (2.75% - 9.43%) (Figure 1).

In contrast, two other co-stimulatory molecules were expressed on the surface from most cells of both A549 cell line and fresh AECII. CD54 was expressed on nearly all the freshly isolated type II AEC (97.37%, 94.88% - 99.55%) and on an important proportion of A549 cells (59.21%, 39.68% - 71.21%), with however significant differences in the proportions of positive cells within the cell suspensions (*P *< 0.01) (Figure 1). Conversely, CD58 was expressed on nearly all the A549 cells (98.54%, 97.70% - 98.89%) and on a lower percentage of freshly isolated cells (58.99%, 52.07% - 73.23%) (*P *< 0.01 ) (Figure 1).

These results indicate the constitutive surface expression of HLA-DR on freshly isolated AECII and the low expression of this molecule on the A549 cell line. The presence of alternative co-stimulatory molecules was highlighted for the two cell suspensions, as well as the very low or even absent expression of the B7 family molecules.

### Modulation of A549 phenotypic markers expression with inflammatory cytokines

In the course of a pulmonary infection, alveolar epithelial cells will be exposed to different inflammatory cytokines released by cells involved in the innate immunity that could modulate the expression of phenotypic markers. We therefore analyzed the expression of these markers in A549 cell line in presence of IFN-γ and/or TNF-α.

**Figure 2:**
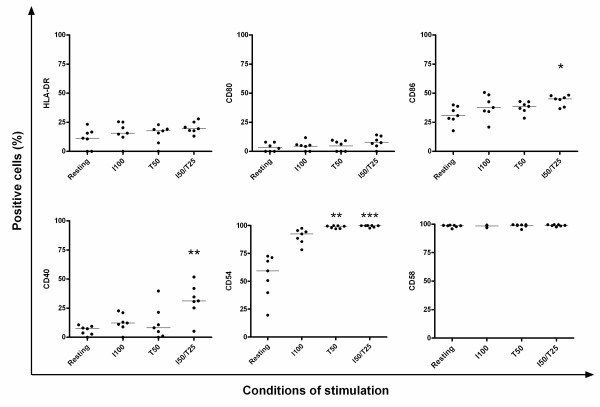
**Modulation of the A549 phenotype by IFN-γ and/or TNF-α (in percentage)**. The A549 cells were incubated during 24 hrs with human recombinant IFN-γ (100 ng/ml, I100), TNF-α (50 ng/ml, T50) or both cytokines (50 ng/ml IFN-γ and 25 ng/ml TNF-α, I50/T25). The phenotype was analyzed by flow cytometry. The horizontal bars represent the medians of the results from seven independent experiments (each performed in triplicate). Mean values and medians are represented as dots and bars respectively. A value of *P <*0.05 was considered to be significant. * *P *< 0.05;** *P *< 0.01; *** *P *< 0.001.

**Figure 3:**
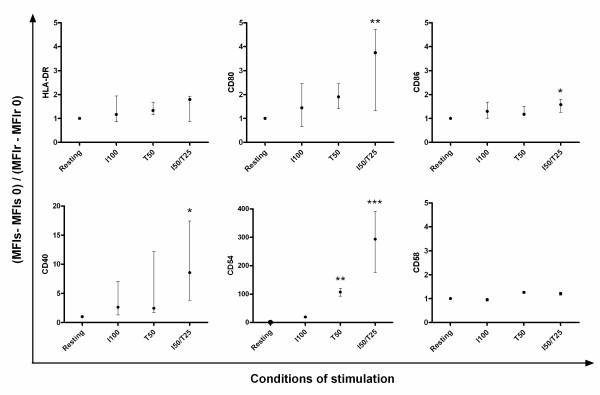
**Modulation of the phenotypic markers density of A549 by inflammatory cytokines**. These results were obtained from the same samples as those presented in Figure 2 but relative medians of fluorescence intensity (MFI) were analyzed. Results are represented by the medians and the inter-quartile ranges obtained for seven independent experiments. A value of *P <*0.05 was considered to be significant. * *P *< 0.05;** *P *< 0.01; *** *P *< 0.001. I100: IFN-γ 100 ng/ml; T50: TNF-α 50 ng/ml; I50/T25: IFN-γ 100 ng/ml, TNF-α 25 ng/ml.

The results illustrated on Figure 2 are expressed as percentages of positive cells and as a ratio between the MFI obtained under stimulating conditions and those obtained on resting cells (Figure 3). The percentages of HLA-DR-expressing cells as well as the HLA-DR and CD58 MFIs did not change after stimulation with IFN-γ and/or TNF-α. In contrast, both the proportion of CD54-positive cells and the CD54-MFI were significantly higher in the presence of TNF-α (*P *< 0.01) and when the cells were cultured with the combination of IFN-γ and TNF-α (*P *< 0.001). The percentage of cells expressing CD80 was not induced by cytokine stimulations, only some increase of the CD80-MFI was noted in the presence of the combination of IFN-γ and TNF-α (*P *< 0.01). Finally, the combined stimulation with IFN-γ and TNF-α increased both the proportion of CD86 and CD40-positive cells and the MFI of these molecules (*P *< 0.05 for CD86 positive cells and CD86 MFI; and *P *< 0.01 and *P *< 0.05 for CD40 positive cells and CD40 MFI respectively).

### Modulation of fresh human AECII phenotypic markers expression with inflammatory cytokines

The effect of IFN-γ and/or TNF-α on the expression of phenotypic markers were also evaluated on freshly isolated AECII as phenotypic differences were observed compared to the A549 cell line.

**Figure 4:**
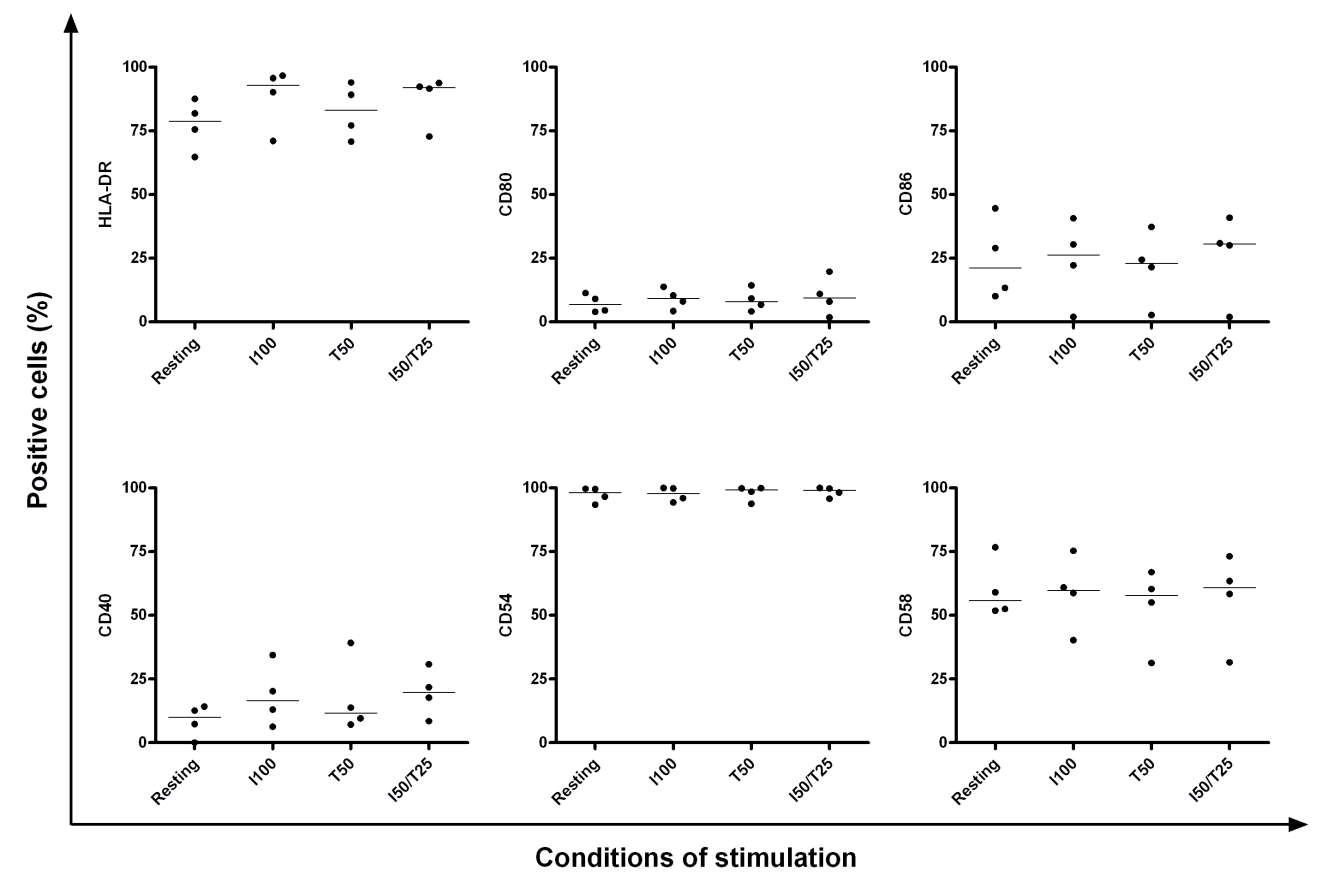
**Modulation of fresh AECII phenotype by IFN-γ and/or TNF-α (in percentage)**. AECII were treated as was A549 cell line in Figure 2. Four independent experiments were performed in triplicate, each point representing the mean value. The bars represent the medians. Results were compared to their resting conditions. I100: IFN-γ 100 ng/ml; T50: TNF-α 50 ng/ml; I50/T25: IFN-γ 100 ng/ml, TNF-α 25 ng/ml.

**Figure 5:**
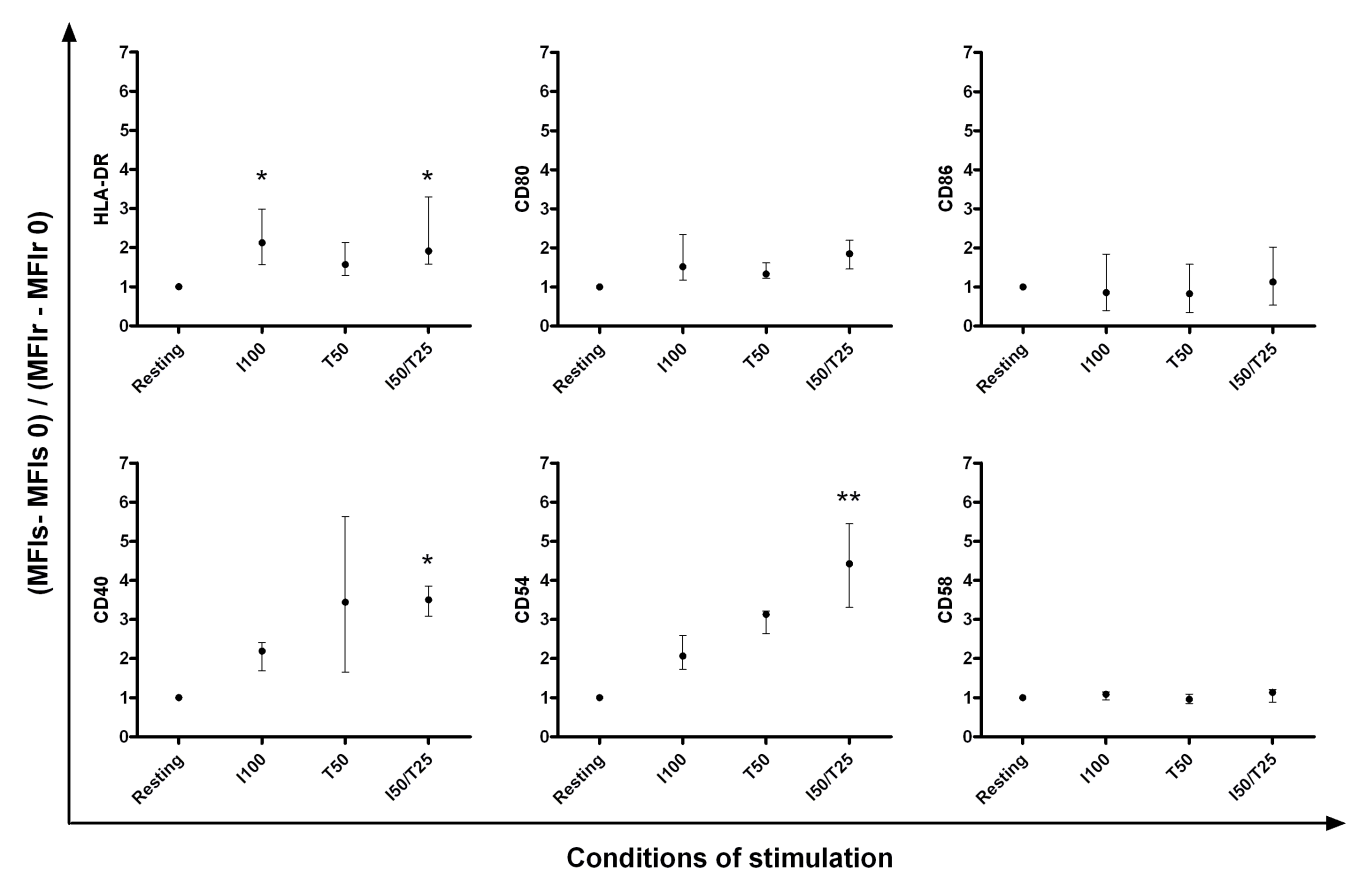
**Modulation of the phenotypic markers density of fresh AECII by inflammatory cytokines**. These results were obtained from the same samples as those presented in Figure 4 but relative medians of fluorescence intensity (MFI) were analyzed. Results are represented by the medians and the inter-quartile ranges obtained with four independent experiments. A value of *P <*0.05 was considered to be significant. * *P *< 0.05;** *P *< 0.01. I100: IFN-γ 100 ng/ml; T50: TNF-α 50 ng/ml; I50/T25: IFN-γ 100 ng/ml, TNF-α 25 ng/ml.

The percentages of type II AEC expressing HLA-DR or the different co-stimulatory molecules investigated here were not different after incubating the cells with IFN-γ and/or TNF-α when compared to the resting cells (Figure 4). In contrast, a slight but significant increase in the MFI of different phenotypic markers was observed in the presence of the inflammatory cytokines indicating that the density of the markers at the cell surface was higher even if the proportion of positive cells did not increase (Figure 5). Higher expression of HLA-DR was observed in the presence of IFN-γ and the combination of cytokines (*P *< 0.05). Similarly, a significantly higher expression of CD54 was noted after incubation of the cells especially with both IFN-γ and TNF-α (*P *< 0.01). The combination of the two cytokines also induced a higher expression of CD40 (*P *< 0.05). No change was noted in the cell surface expression of CD80, CD86 and CD58 molecules, the last one being expressed at basal level by the majority of the cells.

These results on the increase in the expression of CD54 suggest a possible major implication of this molecule in the immunological synapse, without excluding a role for CD58 in co-stimulatory signal transduction.

## Discussion

If the role of AECII in the innate immune system is well recognized, their role as accessory antigen-presenting cells has been more recently proposed. However, until now different contradictory results have been published both concerning the surface-expressed molecules involved in antigen presentation and the *in vitro *T lymphocyte response to AECII-presented antigens [13,15-19,31,32]. We postulated that such differences could be due to the use of different cell types (cell line or not), and to the absence of standardization of the techniques used to analyze these markers. The expression of co-stimulatory molecules by AECII was first reported but recent papers claim that some markers are absent and could contribute to an unresponsiveness of T lymphocytes to AECII stimulation. However, as interstitial lung lymphocytes comprise as much CD8^+ ^and CD4^+ ^lymphocytes [Beukinga I, personal communication], we carefully compared here the classically used model of type II AEC, the A549 cell line, to freshly isolated AECII, for their surface expression of the major markers involved in the immunological synapse. As these surface molecules can be modulated by inflammatory molecules released during lung infections, we further analyzed the modulation of these markers expression by two major inflammatory cytokines, IFN-γ and TNF-α [17,21-26]. We adapted the protocol described by I.R. Witherden and T.D. Tetley for the isolation of AECII as the recommended enzymatic treatment with trypsin does not affect the expression of surface markers on AECII [13], in contrast to dispase sometimes used to isolate AECII and who degrades ICOS-L [18].

The expression of MHC-II molecules is mandatory for the antigen presentation to CD4^+ ^T lymphocytes and we confirmed that freshly isolated AECII have a high constitutive surface expression of HLA-DR molecules as described by Cunningham *et al. *[31] whereas the A549 cell line did not expressed the MHC-II molecules. The MHC-II molecules expression of the latest one was even not modulated by IFN-γ and/or TNF-α. These two last observations reinforce the data obtained by Redondo *et al. *who find a very low expression of MHC-II molecules by the A549 cell line with no modulation by IFN-γ [21]. In contrast, we reported here that the constitutive MHC-II molecules expression by freshly isolated cells was significantly upregulated by IFN-γ and TNF-α, suggesting further the potential of these cells to present antigens to CD4^+ ^T lymphocytes. These data differ from those reported by Debabbi *et al. *[17], most probably as a consequence of differences in the set up analysis used. Indeed, as stressed in the Material and Methods section, we subtracted the auto-fluorescence of non-stained cells before comparing the MFI from stimulating and resting cells to take into account the observed MFI increase of the resting cells, probably secondary to morphological changes.

In addition to the expression of MHC molecules, antigen presentation to lymphocytes usually needs co-stimulatory signals. The most powerful one is given by the B7/CD28 interaction [33]. In agreement with Cunningham *et al.*, we showed here a lack of expression of CD80 (B7-1) and a low expression of CD86 (B7-2) both on the A549 cell line and on the freshly isolated AECII [31]. Only a slight increase in the densities of CD80 and CD86 and in the number of CD86 expressing cells on the A549 cell line was noted in the presence of IFN-γ and TNF-α. Another member of the B7 family, ICOS-L (B7-H2), has been described as playing a role in the activation of memory T lymphocytes [34], but we did not confirm its previously reported expression on AECII [26]. Indeed, we found a very low expression of ICOS-L with no modulation, both on the A549 cell line and on the freshly isolated AECII (data not shown). Globally, these results indicate that B7 family molecules are expressed only at low level by AECII and that their surface expression is not strongly induced upon activation, as opposed to professional APC, such as activated dendritic cells or macrophages. Finally, the expression of CD40 was also shown to be low at the basal state on both cell types with however a slight but significant increase after stimulation with the combination of IFN-γ and TNF-α. Whether the increased expression of these molecules observed in both A549 and AECII, has an impact on the antigenic presentation need to be proved.

All these data suggest that AECII are probably not able to activate neither naive nor memory T lymphocytes by the classical pathway. However, alternative co-stimulatory signals have been reported allowing the stimulation in recall responses of CD4^+ ^and CD8^+ ^T lymphocytes in the absence of B7/CD28 interaction. This pathway involves the CD54/LFA-1 and/or CD58/CD2 interactions [35-37]. Both the A549 cell line and the freshly isolated AECII expressed CD54 and CD58 with a higher level of expression of CD54 and a lower expression of CD58 on the AECII compared to the A549 cell line. Even if no regulation of the expression of CD58 was observed after cytokines stimulation, its high basal expression suggests that this molecule could play a role in the immunological synapse and allow efficient lymphocyte activation as shown for endothelial cells [36]. In contrast, the expression of CD54 was highly upregulated after cytokines stimulation on both cell types. The role of these molecules when the B7/CD28 interaction is lacking was previously shown in a mouse model [35]. Therefore, we suggest that both CD58 and CD54 could play a major role in the antigenic presentation by AECII and that these cells could be able to present antigens to both CD4^+ ^and CD8^+ ^T lymphocytes.

## Conclusions

A549 cell line is not suitable to analyze the antigen presentation to CD4^+ ^T lymphocytes as it lacks the MHC-II surface expression. However, as it kept the expression of MHC-I molecules, as well as the expression of CD54 and CD58, this cell line could be appropriate to study the interactions with CD8^+ ^T lymphocytes. In contrast, freshly isolated AECII could play a role in the activation of both CD4^+ ^and CD8^+ ^T lymphocytes as they expresses MHC-II and MHC-I molecules as well as alternative co-stimulatory CD54 and CD58.

## Competing interests

The authors declare that they have no competing interests.

## Authors' contributions

VC made substantial contributions to the analysis and interpretation of the data, and wrote the manuscript. VD performed experiments, interpreted the results, and critically read the manuscript. SN, MR provided us with macroscopically tumor free regions of lung tissues obtained following lobectomy or pneumectomy for lung cancer performed by MC Thoracic Surgery department. FM planned the concept and study design, critically read and corrected the manuscript. All the authors have critically read the manuscript and approved its submission.
